# Chronic hepatitis C infection–induced liver fibrogenesis is associated with M2 macrophage activation

**DOI:** 10.1038/srep39520

**Published:** 2016-12-21

**Authors:** Moses T. Bility, Kouki Nio, Feng Li, David R. McGivern, Stanley M. Lemon, Eoin R. Feeney, Raymond T. Chung, Lishan Su

**Affiliations:** 1 Department of Infectious Diseases and Microbiology, Graduate School of Public Health, University of Pittsburgh, Pittsburgh, PA, USA; 2 Lineberger Comprehensive Cancer Center, University of North Carolina at Chapel Hill, Chapel Hill, NC, USA; 3 Department of Microbiology and Immunology, University of North Carolina at Chapel Hill, Chapel Hill, NC, USA; 4 Division of Infectious Diseases, Department of Medicine, The University of North Carolina at Chapel Hill, Chapel Hill, NC, USA; 5 University College Dublin, Dublin, Ireland, USA; 6 Massachusetts General Hospital, Boston, MA, USA; 7 Harvard Medical School, Boston, MA, USA

## Abstract

The immuno-pathogenic mechanisms of chronic hepatitis C virus (HCV) infection remain to be elucidated and pose a major hurdle in treating or preventing chronic HCV-induced advanced liver diseases such as cirrhosis. Macrophages are a major component of the inflammatory milieu in chronic HCV–induced liver disease, and are generally derived from circulating inflammatory monocytes; however very little is known about their role in liver diseases. To investigate the activation and role of macrophages in chronic HCV–induced liver fibrosis, we utilized a recently developed humanized mouse model with autologous human immune and liver cells, human liver and blood samples and cell culture models of monocyte/macrophage and/or hepatic stellate cell activation. We showed that M2 macrophage activation was associated with liver fibrosis during chronic HCV infection in the livers of both humanized mice and patients, and direct-acting antiviral therapy attenuated M2 macrophage activation and associated liver fibrosis. We demonstrated that supernatant from HCV-infected liver cells activated human monocytes/macrophages with M2-like phenotypes. Importantly, HCV-activated monocytes/macrophages promoted hepatic stellate cell activation. These results suggest a critical role for M2 macrophage induction in chronic HCV-associated immune dysregulation and liver fibrosis.

Chronic hepatitis C virus (HCV) infection progressively results in chronic liver inflammation, cirrhosis and development of hepatocellular carcinoma (HCC) over several decades^1^. Chronic HCV infection is also associated with impaired immune responses to viral antigens and chronic immune cell infiltration and activation in the liver, leading to progressive liver disease^1^. The development of direct-acting antiviral (DAA) drugs has played a significant role in controlling chronic HCV infection and associated liver disease; however liver fibrosis and HCC development and progression is still a major problem in chronically infected patients^1^. Delineation of the mechanisms by which HCV evades host immunity to establish chronic infection and promote liver disease is a major hurdle in treating chronic HCV-induced advanced liver disease^2^,^3^,^4^. HCV and other human hepatotropic pathogens including HBV have host species restriction, namely humans and chimpanzees^3^. To overcome host species restriction barrier for *in vivo* infection and disease modeling, we recently developed a novel humanized mouse model with both human immune system and liver cells, the AFC8 transgenic mouse transplanted with human CD34+ hematopoietic stem cells and hepatocyte progenitor cells (AFC8-hu HSC/Hep)^5^,^6^. AFC8-hu mice supported HCV infection in the liver and generated human T-cell response to HCV. Chronic HCV infection in the AFC8-hu mice induced chronic liver inflammation and cirrhosis, which correlated with activation of human hepatic stellate cells and expression of human fibrogenic genes^5^.

Chronic liver inflammation and associated liver pathology in chronic HCV infection is characterized by infiltration of various leukocyte populations including activated macrophages^7^,^8^. Several studies show Macrophages play a critical role in modulating host response and tissue pathology, with M1 polarized macrophages promoting Th1 activation and an associated anti-viral response, while M2 polarized macrophages impair Th1 activation and promote tissue fibrosis and neoplasia^9^,^10^,^11^,^12^,^13^. M1 macrophages express co-stimulatory molecules (i.e. membrane bound/soluble CD80, CD86), Th1 cytokines (i.e. IL12, TNFα, IL1β), and iNOS, while M2-like macrophages express scavenger molecules (i.e. membrane bound/soluble CD163, CD206), fibrogenic factors (i.e. TGFβ, PDGF), Th2 cytokines (i.e. IL10) and arginase 1^9^,^10^,^11^,^12^,^13^. This study characterizes macrophage polarization in chronic HCV-induced liver inflammation and associated liver fibrosis in both humanized mice and humans. Additionally, this study characterizes the effect of HCV on macrophage polarization and the role of HCV activated macrophages in hepatic stellate cell activation.

## Results

### HCV-induced liver fibrosis is associated with human M2 macrophage activation in humanized-livers

**Figure 1:**
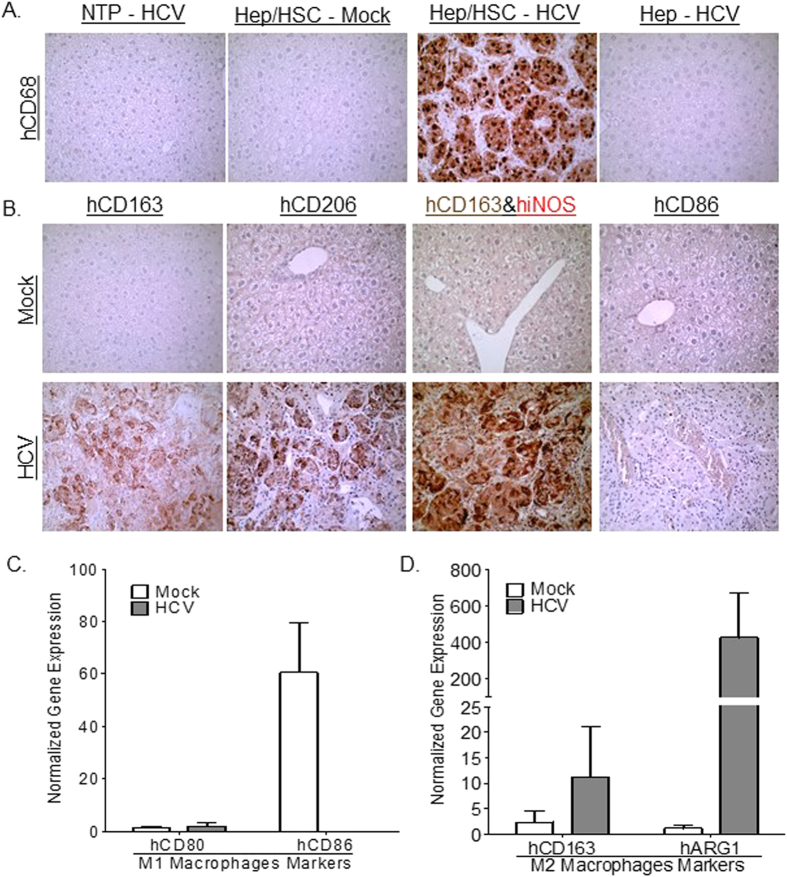
Human infiltrating macrophages express M2 macrophage markers in HCV-infected humanized mice livers. Representative livers of NTP-HCV (Non-transplanted mice inoculated with HCV), Hep/HSC TP - Mock or HCV (humanized mice with human hepatocytes and immune cells inoculated with Mock or HCV+ serum), Hep TP-HCV (humanized mice transplanted with adult hepatocytes inoculated with HCV) were stained for human pan-macrophage marker - hCD68 (**A**) and human M2 (hCD206, hCD163) and M1 (hiNOS, hCD86) markers (**B**). Additionally, liver RNA was isolated from Hep/HSC TP – Mock (N = 3) or HCV (N = 10) and qPCR analysis was performed to examine human (**C**) M1 macrophage polarization markers (hCD80, hCD86) and (**D**) M2 macrophage polarization markers (hARG1, hCD163).

**Figure 2:**
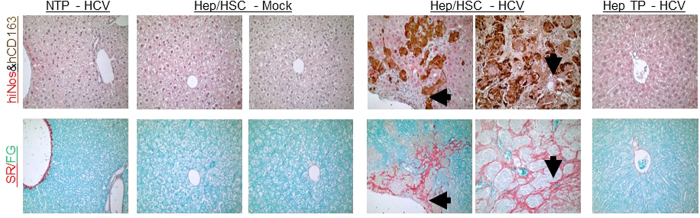
Human infiltrating macrophages localize to fibrotic regions in livers of HCV-infected humanized mice. (**A**) Representative serial liver sections of NTP (Non-transplanted mice inoculated with HCV); Hep/HSC TP - Mock or HCV (humanized mice with human hepatocytes and immune cells inoculated with Mock or HCV+ serum); Hep TP (mice transplanted with human adult hepatocytes inoculated with HCV) were stained for M1 (hiNOS/Red) and M2 (hCD163/Brown) macrophage polarization markers and collagen (Sirius Red/Fast Green). Black arrows denote same regions on serial slides.

Chronic liver inflammation in chronic HCV infection is characterized by infiltration of various leukocyte populations including activated macrophages. Several studies show macrophage polarization plays a critical role in modulating pathogen clearance, chronic inflammation and associated tissue pathology; with M1 polarized macrophages promoting pathogen clearance, while M2 polarized macrophages promote Th1 impairment and tissue fibrosis^14^. Immunohistochemical analysis of chronic HCV-associated liver inflammatory cells in humanized mice showed high levels of macrophages (CD68^positive^) of predominately M2 lineage (CD163^high^, CD206^high^, iNOS^negative^, CD86^negative^) (Fig. 1A,B and Table 1). Additionally, gene expression analysis also showed M2 macrophage polarization (hARG1^high^, hCD163^high^, hCD80^low^, hCD86^low^) is associated with chronic HCV infection in humanized mice (Fig. 1C). Several studies have demonstrated that M2 macrophages secrete immunosuppressive cytokines, inhibitory molecules and lacks co-stimulatory molecules, thus promoting Th1 impairment and viral persistence in chronic viral infections^14^. Additionally, M2 macrophages (alternatively activated macrophages) secrete pro-fibrotic cytokines and factors, thus promoting tissue fibrosis and neoplasia^14^. Histochemical analysis of chronic HCV-induced liver fibrosis in humanized mice showed that M2 macrophages localized to fibrotic regions (Fig. 2). Importantly, chronic HCV-induced liver fibrosis levels correlated with M2 macrophages levels in humanized mice (Table 1).

### HCV-induced liver fibrosis is associated with M2 macrophage activation in the livers of humans

**Figure 3:**
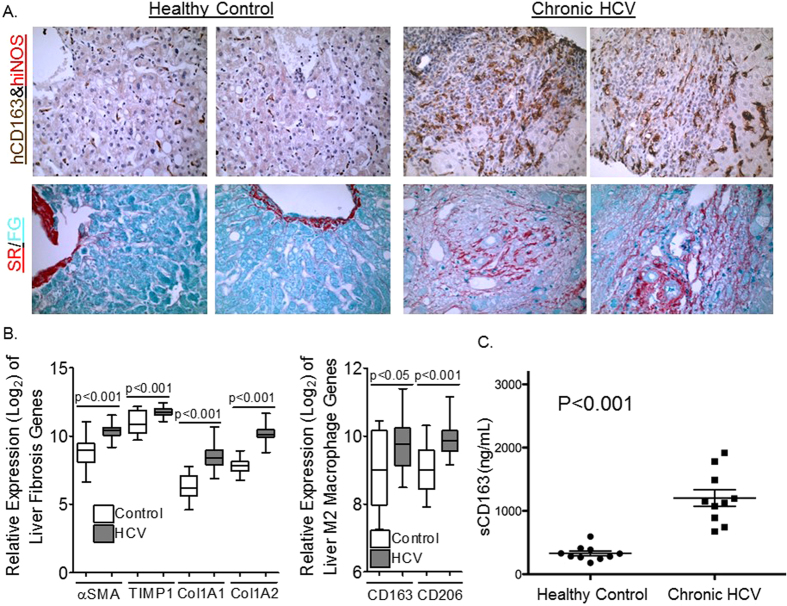
HCV-induced liver fibrosis in humans is associated with infiltrating macrophages expressing M2 macrophage activation markers. (**A**) Representative serial liver sections of healthy controls or chronic HCV infected patients were stained for M1 (hiNOS/Red) and M2 (hCD163/Brown) macrophage and collagen (Sirius Red/Fast Green). (**B**) Chronic HCV-induced liver fibrosis (αSMA, TIMP1, COL1A1, COL1A2) is associated with upregulation of M2 macrophage markers (CD163, CD206) in livers of chronic HCV patients (N = 41) compared to livers of healthy controls (N = 19). (**C**) Elevated serum levels of soluble M2 macrophage activation marker (Soluble CD163) in chronic HCV patients with liver fibrosis (N = 10) compared to healthy controls (N = 10).

To confirm results from the humanized mouse model, livers from healthy controls and patients with chronic HCV–induced advanced liver disease (cirrhosis) were examined (Table 2). Analysis of chronic HCV-induced liver inflammatory cells in humans showed elevated levels of M2 macrophages (CD163^high^, iNOS ^negative^) (Fig. 3A). Importantly, M2 macrophages in infected livers localized to fibrotic regions (Fig. 3A). Additionally, analysis of published gene microarray dataset of livers from healthy controls without liver disease and chronic HCV patients with cirrhosis and end-stage liver disease (Table 2) showed upregulation of hepatic stellate cell activation (αSMA, TIMP1, COL1A1 and COL1A2 mRNA levels) genes was associated with upregulation of M2 macrophage genes (CD163 and CD206 mRNA levels) in chronic HCV infected livers compared to healthy control livers^15^ (Fig. 3B). Analysis of serum from healthy controls and chronic HCV infected patients with liver disease showed chronic inflammation in HCV infection is associated with elevated M2 macrophage activation as examined using soluble CD163 levels (M2 macrophage activation marker) (Fig. 3C); soluble CD80 levels (M1 macrophage activation marker) were not detectable. This result is in concordance with recently published studies demonstrating soluble CD163 and soluble MRC1 (M2 macrophage activation markers) levels correlate to the severity of chronic C hepatitis virus–induced liver fibrosis^16^,^17^.

### Direct-acting antiviral (DAA) therapy is associated with reduced M2 macrophages and liver disease in chronic HCV infected patients

**Figure 4:**
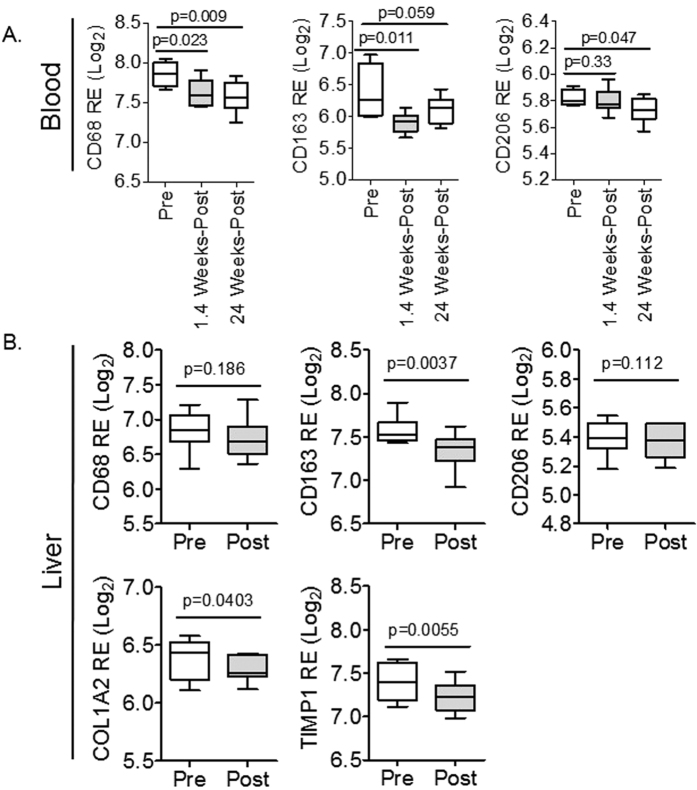
Direct-acting antiviral (DAA) therapy is associated with reduced M2 macrophage activation genes and liver disease in chronic HCV-infected patients. (**A**) Direct-acting antiviral (DAA) therapy in chronic HCV patients is associated with reduced macrophage (CD68) and M2 macrophage markers (CD163, CD206) in peripheral blood mononuclear cells (PBMCs) after 1.4 weeks (N = 8) and 24 weeks of therapy (N = 12), when compared to pre-treatment (N = 4). (**B**) DAA therapy results in attenuation of liver fibrogenesis (COL1A2, TIMP1), associated with significantly reduced CD163 mRNA levels in the liver, as examined in paired chronic HCV patients biopsies prior to (N = 8) and after (N = 8) treatment regimen (24 weeks of DAA therapy).

To further analyze the nexus between M2 macrophages and liver fibrogenesis in chronic HCV infection, the effect of anti-viral treatment on M2 macrophage levels was examined in chronic HCV patients with liver disease and undergoing treatment with direct-acting antiviral (DAA) plus Ribavirin (Table 2)^18^. Analysis of PBMCs prior to and after treatment (1.4 weeks and 24 weeks of therapy) with DAA (Sofosbuvir plus Ribavirin) showed virological response (prior to any virological relapse – approximately 10% of patients relapsed) is associated with reduced monocyte/macrophage (CD68 mRNA level) and M2 macrophage markers (CD163 and CD206 mRNA levels) in PBMCs (Fig. 4A)^18^. Also, analysis of paired chronic HCV infected human livers prior to and end of treatment (24 weeks of therapy) with DAA (Sofosbuvir plus Ribavirin) (Table 2) show virological response (prior to any virological relapse) and attenuation of liver fibrogenesis (reduced TIMP1 and COL1A2 mRNA levels) is associated with significantly reduced CD163 mRNA levels in the liver; albeit no significant differences was observed in CD68 and CD206 mRNA levels (Fig. 4B)^18^.

### HCV induces M2 activation in monocytes and macrophages

**Figure 5:**
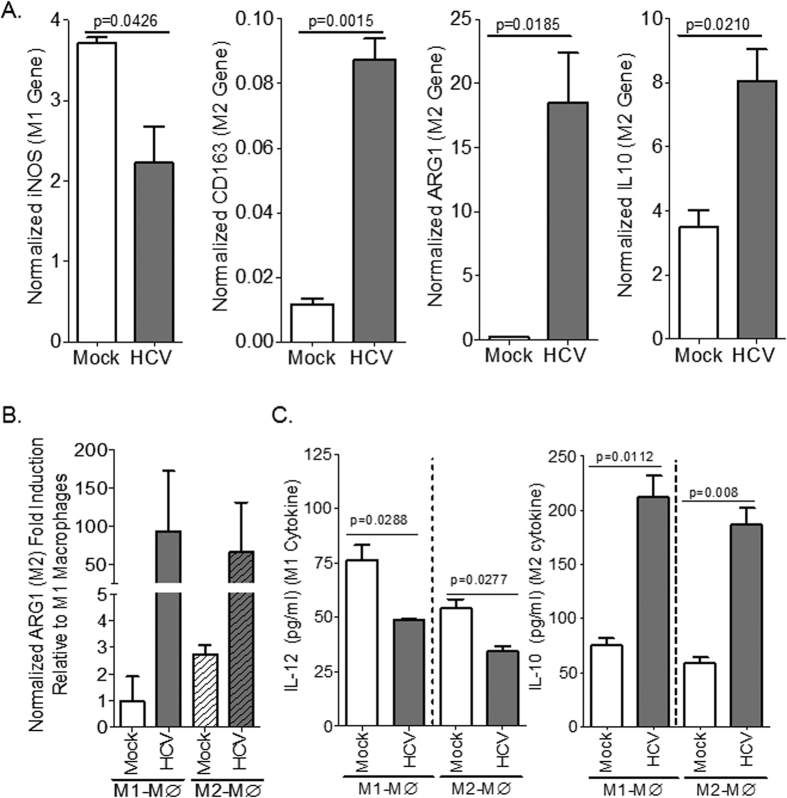
HCV induces M2 activation in monocytes and macrophages. (**A**) Human monocytic cells (THP1) were inoculated with supernatant from JFH1/genotype 2-infected Huh7.5 cells or mock-infected Huh7.5 cells and macrophage polarization was examined using gene expression analysis after 6 days. (**B,C**) Primary human monocytes were differentiated to M1 or M2 macrophages and were inoculated with supernatant from JFH1/genotype 2-infected Huh7.5 cells or mock-infected Huh7.5 cells and macrophage polarization markers were examined using gene expression (**B**) and cytokine (**C**) analysis after 6 days.

Intracellular pathogens such as viruses stimulate M1 macrophage response and associated Th1 immune response in the infected host, which results in acute infection, virus control/clearance and host immunity; however, Th1 response is impaired in chronic viral infections^14^. Results from several studies suggest that HCV promotes immune impairment and immunosuppressive phenotype in monocytes/macrophages^19^,^20^,^21^,^22^. In this study we showed that culturing human THP1 monocytic cell line and monocytes with supernatant from HCV-infected Huh7.5 cells resulted in induction of M2-like activation when compared to supernatant from mock-infected cells, as measured by elevated CD163, ARG1, IL10 and reduction of iNOS gene expression levels (Fig. 5A). Additionally, culturing M1 (GM-CSF) or M2 (MCSF)-polarized macrophages with supernatant from HCV-infected Huh7.5 cells resulted in induction of M2 macrophage activation when compared to supernatant from mock-infected cells, as measured by elevated ARG1 gene expression and IL10 cytokine level, and reduction of M1 macrophage activation as measured by reduced IL12 cytokine level (Fig. 5B,C).

### HCV-activated monocytes/macrophages promote hepatic stellate cell activation

**Figure 6:**
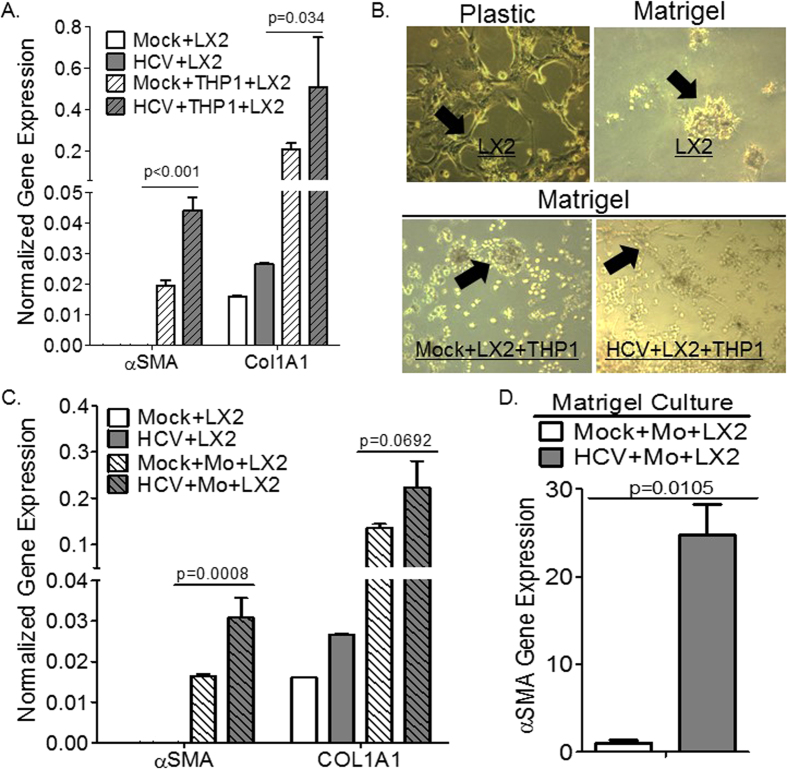
Human macrophages exposed to HCV promote hepatic stellate cell activation. (**A**) Human monocytic cells (THP1) were cultured with supernatant from H77S.3/genotype 1 or JFH1/genotype 2-infected Huh7.5 cells or mock-infected Huh7.5 cells for 6 days, and subsequently co-cultured with human hepatic stellate cells (LX2) on plastic plates for 24 hours; fibrotic gene expression was examined by qPCR. (**B**) Human hepatic stellate cells (LX2 cells) cultured on Matrigel coated plates to induce cellular quiescence, were subsequently co-cultured with human monocytic cell line (THP1 cells) and supernatant from JFH1/genotype 2-infected Huh7.5 cells or from mock-infected Huh7.5 cells, and hepatic stellate cells activation was examined morphologically after 3 days (black arrows: quiescent cells phenotype - round clumps; while activated phenotype - fibroblast-like shape). (**C**) Primary human monocytes were cultured with supernatant from JFH1/genotype 2-infected Huh7.5 cells or mock-infected Huh7.5 cells for 6 days, and subsequently co-cultured with human hepatic stellate cells (LX2) on plastic plates for 24 hours; fibrotic gene expression was examined by qPCR. (**D**) Human hepatic stellate cell line (LX2), cultured on Matrigel coated plates to induce quiescence, were subsequently co-cultured with primary monocytes and supernatant from H77S.3/genotype 1-infected Huh7.5 cells or mock-infected Huh7.5 cells and hepatic stellate cell activation markers was analyzed after 3 days by gene expression.

As mentioned earlier, several studies have suggested that macrophage polarization plays a critical role in tissue remodeling and pathology; with M2 macrophage directly promote tissue fibrosis via the production of pro-fibrogenic factors and molecules. Hepatic stellate cell activation is a highly regulated biological process with critical roles for parenchymal cells (hepatocytes), non-parenchymal cells (i.e. monocytes and macrophages), and the stroma^23^. Here, we examined the effect of HCV activated monocytes/macrophages on hepatic stellate cell activation using human hepatic stellate cell line (LX2 cell line), and different stroma conditions to mimic physiological conditions. Co-culturing human hepatic stellate cells (LX2) with HCV-activated monocytic cells (THP1) in plastic stroma conditions resulted in upregulation of well-established hepatic stellate cell activation markers compared to mock control, as examined using gene expression analysis (αSMA and COL1A1) (Fig. 6A). Supernatant from HCV-infected Huh7.5 cells had negligible effect on LX2 cell activation when compared to supernatant from mock-infected cells (Fig. 6A). Here we also confirmed the critical role of the stroma in modulating hepatic stellate cell activation state; culturing hepatic stellate cells (LX2 cell line) on Matrigel coated plates induced cellular quiescence when compared to plastic plates as examined using morphological analysis (Fig. 6B), and gene expression analysis (Data not shown). HCV (JFH1-Huh7.5 supernatant) inoculation of human monocytes (THP1 cell line) and LX2 cells co-cultured in Matrigel coated plates resulted in hepatic stellate cell activation when compared to mock control as examined using morphological analysis, with quiescent LX2 cells exhibiting the characteristic round clump appearance, while activated LX2 cells exhibiting the well-established characteristic star-like morphology (Fig. 6B). We confirmed those results in co-cultures of primary human monocytes/macrophages and hepatic stellate cells. To examine the effect of HCV-activated primary monocytes on hepatic stellate cells activation, HCV (genotype 2 – JFH1)-activated primary monocytes were co-cultured with LX2 cells on plastic plates. Co-culturing HCV-activated primary monocytes with LX2 cells resulted in upregulation of hepatic stellate cell activation markers compared to mock control, as examined using gene expression analysis (αSMA and COL1A1) (Fig. 6C); HCV supernatant had negligible effect on the expression of hepatic stellate cell activation markers when compared to mock supernatant (Fig. 6C). Additionally, co-culture of quiescent LX2 cells and primary monocytes in Matrigel coated plates with HCV (genotype 1 – H77S.3) supernatant also resulted in upregulation of hepatic stellate cell activation markers when compared to mock control as examined using gene expression analysis (αSMA) (Fig. 6D).

## Discussion

Chronic hepatitis C virus (HCV) infection affects approximately 2–3% of the global population and cause severe morbidity and mortality, due to progressive liver inflammation, cirrhosis and hepatocellular carcinoma (HCC)^1^. The recent introduction of novel direct-acting anti-viral (DAA) therapies have demonstrated robust clearance of chronic HCV infection across several genotypes, however DAA treatment does not directly target cirrhosis and HCC^1^. Additionally, several challenges in HCV therapies remains, including drug resistant HCV strains, advanced liver disease (cirrhosis and HCC), and the cost of care^1^. Therefore, elucidating the mechanisms by which chronic HCV infection induces liver disease is a major hurdle in treating HCV-induced advanced liver disease such as cirrhosis^3^. Chronic liver inflammation is a hallmark of chronic HCV infection and is characterized by infiltration of a myriad of immune cells including macrophages^7^,^8^. Macrophages are innate immune cells with critical roles in host defense, immune response, tissue maintenance and tissue repair^14^. Macrophages are broadly delineated into two categories, namely M1 macrophages and M2 macrophages^14^. Importantly, several reports suggest macrophage polarization plays a critical role in modulating pathogen clearance, chronic inflammation and associated tissue pathology; with M1 polarized macrophages promoting anti-viral Th1 immune response and viral clearance, while M2 polarized macrophages impair Th1 immune response and promote tissue inflammation and fibrosis^14^. M2 macrophages play critical roles in wound repair, secreting anti-inflammatory cytokines and redistributing micronutrients during repair following injury; however, during chronic infection, M2 macrophages promote tissue fibrosis, cancer development and impair Th1 response, thus promoting pathogen persistence and associated tissue pathology^14^.

In order to understand the mechanism by which chronic HCV induces liver disease, we examined the relationship between macrophage polarization and liver disease, specifically cirrhosis. In this report we demonstrated that liver inflammation and cirrhosis in chronic HCV infected humanized mice livers is associated with M2 macrophages. HCV-induced M2 macrophages localized to fibrotic regions in HCV-infected livers in humanized mice. Importantly, results from chronic HCV infected patients confirmed that chronic HCV infection in the liver is associated with M2 macrophage accumulation. Additionally, we demonstrated that chronic HCV infection and associated liver disease is also associated with levels of M2 macrophage activation marker in the serum. We also demonstrated that DAA treatment-induced virological response and liver fibrogenesis attenuation is associated with attenuation of M2 macrophages both in the blood and the liver. We demonstrated that supernatant from HCV infected hepatocytes promotes M2 macrophage polarization in human monocytic cells. Additionally, supernatant from HCV infected hepatocytes promotes M2 macrophage polarization in both human M1 and M2 polarized macrophages. Importantly, we provide evidence that suggests HCV-activated monocytes/macrophages promote hepatic stellate cell activation in tissue culture models.

There are several limitations in this study that will be address in future studies. Future *in vivo* mechanistic studies will examine the role of M2 macrophages in promoting and maintaining liver fibrosis in HCV infection via targeting M2 macrophages using small molecules and macrophage depleting agents. Additionally, future studies will address the mechanism by which HCV promotes M2 macrophage activation, and the mechanism by which HCV-activated monocytes/macrophages promote hepatic stellate cell activation. Results from this study suggest that the hepatitis C virus could mediate M2 macrophage activation in human monocytes/macrophages. Several studies have demonstrated a critical role for macrophages in liver fibrosis, with soluble factors such as TGF-B, PDGF, VEGF as potent mediators of macrophage pro-fibrotic activity. Future studies will investigate the expression of pro-fibrotic factors such as TGF-B, PDGF, VEGF in HCV-activated macrophages and their role of hepatic stellate cell activation. A limitation of this study is that we did not extensively characterized HCV-induced M2 macrophages, and the possibility that those cells could acquire myofibroblast-like characteristics and deposit collagen, which are properties of mesenchymal-lineage cells (i.e. activated hepatic stellate cells). Future studies will address the possibility of monocyte/macrophage to mesenchymal transition in HCV-induced M2 macrophages, and the ability of those cells to express well-established markers of hepatic stellate cell activation and promote fibrogenesis. Future studies will also address the relationship between HCV viral load and monocyte/macrophage activation, and the effect of viral clearance on macrophage activation in the liver and the effect on advance liver disease such as cirrhosis and hepatocellular carcinoma. The relationship between M2 monocyte/macrophage activation in the blood and liver is also a topic of significant interest, and could provide insight unto the mechanism of M2 macrophage-associated liver inflammation and the mechanism of liver disease; future studies will address the mechanism of monocyte/macrophage trafficking in the inflamed-livers in humanized mice.

Our results are consistent with recently published studies showing chronic liver infections (chronic hepatitis B; chronic Opisthorchis viverrini infection) and associated hepatobiliary fibrosis and cancer is associated with M2 macrophages^24^,^25^. Chronic HBV infection-induced liver fibrosis in both humans and humanized mice is associated with M2 macrophages, which are localized to fibrotic regions^24^. Importantly, acute HBV-induced accelerated liver fibrosis and failure is also associated with upregulation of M2 macrophage genes, and downregulation of M1 macrophage genes^24^ using published gene microarray dataset^26^,^27^. Additionally, chronic Opisthorchis viverrini (OV) infection-induced liver fibrosis in both humans and Syrian golden hamsters was associated with M2 macrophages, which localized to fibrotic regions and neoplastic regions^25^. Those results and this study suggest a possible underlying mechanism by which liver pathogens (i.e. HCV, HBV, and OV) and other non-infectious agents (i.e. high fat diet), along with co-morbidities (i.e. HIV co-infection) modulate host macrophage polarization to promote hepatobiliary fibrogenesis and carcinogenesis.

In summary, this study sheds light on the role of M2-like macrophages in modulating liver fibrosis in chronic HCV infection. Importantly, we showed that HCV infection-induced M2 macrophage activation in the liver may be critical for the induction of hepatic fibrogenesis (Fig. 6). Results from this study suggest a critical role for “M2-like” macrophages in chronic HCV-induced immune impairment, immune dysregulation and liver pathology; suggesting a novel cellular target for HCV therapeutics development. Results from this study also demonstrate that the humanized mouse model and cell culture models will provide a novel mechanistic and pre-clinical platform for evaluating therapeutics targeting human macrophages in chronic HCV infection and its associated liver diseases.

## Methods

### Ethics statement

All animal experiments were conducted following NIH guidelines for housing and care of laboratory animals and in accordance with protocols approved by the institution’s Institutional Animal Care and Use Committee (protocol number 10–107) at the University of North Carolina at Chapel Hill. Liver gene expression profile analyses in patients were obtained from a dataset in Gene Expression Omnibus (GEO)/NCBI database; the reports followed NIH research ethics guidelines. Harvard/MGH Research and Ethics Committee and the independent ethics committee approved human study protocols; written informed consent was obtained from all participants.

### Analysis of HCV-induced liver fibrosis and macrophage polarization in humanized mice and humans

AFC8 liver and immune system - humanized mice or non-humanized control mice were inoculated *iv* with clinical isolates of HCV^5^. At termination, humanized liver tissues were immediately fixed in formalin or frozen. Additionally, liver tissue samples were collected from healthy controls (HCV seronegative individuals with normal liver function) and HCV seropositive patients with liver fibrosis and abnormal liver function and fixed in formalin; serum samples were also collected from those patients and stored at −80 degree Celsius. All animal and human protocols were approved and followed institutional and national guidelines. Fixed tissues were paraffin embedded and sections were stained with Sirius red/fast green (liver fibrosis) or with indicated antibodies. Immunoreactivity was determined by incubation with DAB substrate (Pierce) or Vulcan red (Dako), and counterstained with hematoxylin^28^,^29^. Gene expression was examined using qPCR and Sybr Green kit, following manufacturer’s recommended procedures (Invitrogen). Microarray gene expression analysis was performed on publicly available microarray dataset from healthy controls without liver disease and chronic HCV patients with cirrhosis^15^,^30^ (GEO accession: GSE14323), and from chronic HCV patients undergoing direct-acting antiviral therapy^18^,^31^ (GEO accession: GSE51699) using GEOR (NCBI Software). For GSE14323 microarray studies, livers from adult HCV RNA positive patients with advanced liver fibrosis and undergoing liver transplantation due to end-stage liver disease were obtained via the Adult Living Donor Liver Transplant Cohort Study (NCT00096733) (Table 2); livers from adult HCV RNA negative liver transplant donors with normal liver function and histopathology were obtained through the Liver Tissue Cell Distribution System (Table 2)^15^. For GSE51699 microarray studies, blood and liver samples from adult HCV genotype-1 RNA positive patients, some with advanced liver fibrosis (20%) as measured using Knodell-HAI scores by the study investigators were obtained as part of a clinical trial for direct-acting antiviral (DAA) (NCT01441180) (Table 2). DAA reduced viral load in all patients, as documented by reduction below lower limit of detection (LLOD) of 3 IU/ml. Samples were collected prior to viral relapse, which occurred in a minority of patients (10%)^18^. Soluble CD163 and soluble CD80 were examined in serum samples of adult healthy controls (HCV RNA negative individuals with normal liver function) and HCV RNA positive patients with liver fibrosis and abnormal liver function using ELISA and following manufacturers recommended procedures (R&D systems).

### Cell culture models for monocyte/macrophage and hepatic stellate cell activation

Human PBMCs were isolated from buffy coats of healthy HIV/HBV/HCV sero/qPCR negative blood donors by Ficoll-paque density gradient centrifugation. Cells were then washed, suspended in RPMI containing pen/strep (1%), glutamine (1%), heat-inactivated FBS (10%), and seeded on tissue culture plates. To isolate primary monocytes, non-adherent cells were removed by gentle pipette aspiration after 1.5 h of incubation at 37 °C in a humidified atmosphere containing 5% CO_2_. Equal volume of fresh complete medium was then added to each flask and attached cells (primary monocytes) at approximately 70–80% confluency were cultured for 6 additional days at 37 °C in 5% CO_2_ in presence of either rHuGM-CSF (100 ng/ml) (M1 macrophages) or rHuM-CSF (100 ng/ml) (M2 macrophages) for differentiation into polarized M1 or M2 - monocyte derived macrophages. THP1 monocytes and monocyte-derived primary M1 or M2 macrophages were washed and treated with supernatant containing HCV (supernatant from JFH1/genotype 2-infected Huh7.5 cells at 0.5 virus particles per monocytes/macrophages) or Mock (supernatant from mock-infected Huh7.5 cells) for 6 days. M1 and M2 macrophage activation were examined using cytokine analysis (BD Biosciences) and qPCR (Invitrogen) following manufactures’ recommended procedures. For co-culture experiments examining the effect of HCV-activated monocytes on hepatic stellate cell activation, human monocytic cells (THP1) or primary monocytes were cultured with supernatant containing HCV (supernatant from H77S.3/genotype 1 or JFH1/genotype 2-infected Huh7.5 cells at 0.5 virus particles per monocytes/macrophages) or Mock (supernatant from mock-infected Huh7.5 cells) for 6 days; subsequently, HCV or mock-stimulated monocytes were co-culture with human hepatic stellate cells (LX2) at equal proportion on plastic surface for an additional 24 hours and hepatic stellate cell activation was examined using qPCR (Life Technologies). Additionally, human monocytic cells (THP1) were inoculated with supernatant containing HCV (supernatant from JFH1/genotype 2-infected Huh7.5 cells at 0.5 virus particles per monocytes/macrophages) or Mock (supernatant from mock-infected Huh7.5 cells) and co-cultured with human hepatic stellate cells at equal proportion on Matrigel (BD Biosciences) and hepatic stellate cell activation were examined using qPCR (Life Technologies) after 3 days.

### Statistical analysis

We used unpaired or paired two-tailed Student’s t-tests or ANOVA for comparisons. p < 0.05 is considered significant. All data are reported as means ± standard error.

## Additional Information

**How to cite this article**: Bility, M. T. *et al*. Chronic hepatitis C infection–induced liver fibrogenesis is associated with M2 macrophage activation. *Sci. Rep.* **6**, 39520; doi: 10.1038/srep39520 (2016).

**Publisher's note:** Springer Nature remains neutral with regard to jurisdictional claims in published maps and institutional affiliations.

## Figures and Tables

**Table 1:** HCV–induced liver inflammation and fibrosis is associated with M2 macrophage activation in the liver in the AFC8 humanized mouse model.

ID	Model	Inoculum	Time Post Inoculation	Liver Viral Load^*a*^	Liver Disease^*b*^	Liver M2 MØ
1	Non-hu-mice	Mock (PBS)	11.4 Weeks	ND	F0	—
117	HSC-Hep hu-mice	Mock (Human Sera)	6.8 Weeks	ND	F0	—
88*4	HSC-Hep hu-mice	Mock (Human Sera)	13.8 Weeks	ND	F0	—
99*4	HSC-Hep hu-mice	Mock (Human Sera)	13.8 Weeks	ND	F0	—
4	Non-hu-mice	HCV GT1 #1 (9E5 copies/mL)	15.7 Weeks	ND	F0	—
110	Adult Hep hu-mice	HCV GT1 #1 (9E5 copies/mL)	15.7 Weeks	0.18E5 copies/ug RNA	F0	—
108	HSC-Hep hu-mice	HCV GT1 #1 (9E5 copies/mL)	4.6 Weeks	1.64E5 copies/ug RNA	F3	+++
109	HSC-Hep hu-mice	HCV GT1 #1 (9E5 copies/mL)	11.4 Weeks	0.14E5 copies/ug RNA	F3	+++
90*4	HSC-Hep hu-mice	HCV GT1 #2 (5E5 copies/mL)	4.8 Weeks	3.23E5 copies/ug RNA	F3	+++
91*4	HSC-Hep hu-mice	HCV GT1 #2 (5E5 copies/mL)	8.1 Weeks	1.68E5 copies/ug RNA	F2	++
92*4	HSC-Hep hu-mice	HCV GT1 #2 (5E5 copies/mL)	13.8 Weeks	ND	F4	+++

Notes: ID = Identification; HSC-Hep hu-mice = AFC8-hu HSC/Hep mouse; Adult Hep hu-mice = AFC8-hu Adult hepatocytes mouse; Non-hu mice = Non transplanted AFC8 mouse; Inoculum=Mock (PBS or Sera from healthy control humans), HCV#1 (HCV positive serum from patient #1), HCV#2 (HCV positive serum from patient #2), ^*a*^Chronic HCV = Persistent HCV infection markers (HCV RNA) detected in animal as reported in Washburn *et al*.^5^; ^*b*^Liver disease = METAVIR score (Liver fibrosis stage and necroinflammatory activity) as reported in Washburn *et al*. no liver disease (F0), mild liver fibrosis (1), moderate liver fibrosis (2), severe liver fibrosis (>3); Liver M2 MØ = Relative M2-like (hCD163+) macrophage levels, no M2 macrophages (−), low M2 macrophage level (+), intermediate M2 macrophage level (++), high M2 macrophage level (+++).

**Table 2:** HCV–induced liver inflammation and fibrosis is associated with M2 macrophage activation in the liver humans.

GEO NCBI ID	Clinical Trial Identifier	Infection Status	Age	Liver disease Score	Treatment	Outcome
GSE14323	LTCDS^a^	Uninfected	Adult	Normal	NA	NA
GSE14323	NCT00096733	HCV^b^	50.1 (10.9)^c^	14.9 (5.7) MELD	NA	End-stage liver disease
GSE51699	NCT01441180	HCVgt1	54 (50, 57)^d^	0-1 (80%)3-4 (20%) Knodell-HAI	SOF+RBV (24Weeks)	SVR (90%) Relapsed (10%)

Notes: ID = Identification; Liver Tissue Cell Distribution System (LTCDS; a = Not Clinical Trial); b = Includes all HCV genotypes; c = Mean (Standard Deviation) (Olthoff, K.M *et al*.^30^), d = Median (Interquartile range) (Osinusi, A. *et al*.^31^); Model For End-Stage Liver Disease (MELD) and standard deviation (#); Knodell Histology Activity Index (Knodell-HAI) and percentage with score range (%); NA = Non-applicable; SVR = sustained virologic response.
